# Enhancing Acute Migraine Treatment: Exploring Solid Lipid Nanoparticles and Nanostructured Lipid Carriers for the Nose-to-Brain Route

**DOI:** 10.3390/pharmaceutics16101297

**Published:** 2024-10-04

**Authors:** Joana Torres, Renata Silva, Gonçalo Farias, José Manuel Sousa Lobo, Domingos Carvalho Ferreira, Ana Catarina Silva

**Affiliations:** 1 UCIBIO, Laboratory of Pharmaceutical Technology, Faculty of Pharmacy, University of Porto, 4050-313 Porto, Portugal; 2 Associate Laboratory i4HB Institute for Health and Bioeconomy, Faculty of Pharmacy, University of Porto, 4050-313 Porto, Portugal; 3 UCIBIO, Laboratory of Toxicology, Department of Biological Sciences, Faculty of Pharmacy, University of Porto, 4050-313 Porto, Portugal; 4 Aptar Pharma, 27100 Le Vaudreuil, France; 5 FP-BHS (Biomedical and Health Sciences Research Unit), FP-I3ID (Instituto de Investigação, Inovação e Desenvolvimento), Faculty of Health Sciences, University Fernando Pessoa, 4200-150 Porto, Portugal

**Keywords:** acute migraine, lipid nanoparticles, solid lipid nanoparticles (SLN), nanostructured lipid carriers (NLC), nose-to-brain, co-encapsulation

## Abstract

Migraine has a high prevalence worldwide and is one of the main disabling neurological diseases in individuals under the age of 50. In general, treatment includes the use of oral analgesics or non-steroidal anti-inflammatory drugs (NSAIDs) for mild attacks, and, for moderate or severe attacks, triptans or 5-HT_1B/1D_ receptor agonists. However, the administration of antimigraine drugs in conventional oral pharmaceutical dosage forms is a challenge, since many molecules have difficulty crossing the blood-brain barrier (BBB) to reach the brain, which leads to bioavailability problems. Efforts have been made to find alternative delivery systems and/or routes for antimigraine drugs. In vivo studies have shown that it is possible to administer drugs directly into the brain via the intranasal (IN) or the nose-to-brain route, thus avoiding the need for the molecules to cross the BBB. In this field, the use of lipid nanoparticles, in particular solid lipid nanoparticles (SLN) and nanostructured lipid carriers (NLC), has shown promising results, since they have several advantages for drugs administered via the IN route, including increased absorption and reduced enzymatic degradation, improving bioavailability. Furthermore, SLN and NLC are capable of co-encapsulating drugs, promoting their simultaneous delivery to the site of therapeutic action, which can be a promising approach for the acute migraine treatment. This review highlights the potential of using SLN and NLC to improve the treatment of acute migraine via the nose-to-brain route. First sections describe the pathophysiology and the currently available pharmacological treatment for acute migraine, followed by an outline of the mechanisms underlying the nose-to-brain route. Afterwards, the main features of SLN and NLC and the most recent in vivo studies investigating the use of these nanoparticles for the treatment of acute migraine are presented.

## 1. Introduction

Migraine is considered the most disabling neurological disorder in people under the age of 50 and is characterized by a complex neurobiology involving the central and peripheral nervous systems [1,2,3].

The mechanisms underlying the development of migraine are complex, and the general symptoms include moderate/severe headache attacks associated with symptoms of nausea, vomiting, photo-, and phonophobia. Depending on the intensity and disability of these symptoms, migraine can be considered episodic or chronic. Patients with chronic migraine have greater disability in comparison to patients with episodic migraine. Therefore, current studies on migraine focus mainly on the treatment of episodic migraine, in order to decrease the intensity and frequency of attacks, and to avoid their chronification [4].

Current treatment for migraine includes acute and preventive drugs, which are frequently inefficient in stopping or reducing migraine attacks [4,5]. Most acute therapies used to treat migraine are administered orally, which can have pharmacokinetic limitations related to extensive first-pass metabolism, slow onset of action and difficulty crossing the blood-brain barrier (BBB). In addition, gastrointestinal symptoms (i.e., vomiting) that migraineurs often experience can lead to the loss of the administered drugs and uncertainty about whether to re-dose [6,7,8,9]. Thereby, all these limitations have led to the study of new non-invasive approaches that are safe and effective to increase patient compliance [9]. In this context, drug administration through the intranasal (IN) route has been described as more effective compared to the oral route, because it is easy to use, provides a faster onset, avoids degradation in the gastrointestinal tract and first-pass hepatic metabolism, shows a decreased adverse effects (AEs), and allows the direct access of the drugs to the central nervous system (CNS), avoiding the need to pass the BBB [8,10].

Although the IN route is promising, the physicochemical characteristics of the drug molecules, the physiological and anatomical properties of the nasal cavity and the characteristics of the formulation can reduce drug absorption in the nasal mucosa. These factors should be taken into account when developing IN formulations, in order to achieve more effective targeting of drugs to the brain [9,11,12,13]. In this regard, the use of nanoparticle-based systems has shown a remarkable ability to overcome these difficulties, promoting the accumulation of drugs in the CNS, thus avoiding their systemic distribution [9,14]. In particular, formulations based on solid lipid nanoparticles (SLN) and nanostructured lipid carriers (NLC) provide various advantages to accomplish with this aim. These nanoparticles enable more efficient transport of the drug from the nose to the brain, increasing absorption and bioavailability. Furthermore, their solid matrix protects the encapsulated drug, minimizing degradation resulting from enzymatic activity in the nasal cavity [15,16].

This review aims to describe the state of the art of using SLN and NLC as a smart approach to improve the treatment of acute migraine via the nose-brain route.

## 2. Acute Migraine

Migraine is a complex disorder recognized by characteristic symptoms, including intense headaches (most unilateral), nausea, and sensitivity to sound and/or light [17]. This disorder has a high socioeconomic impact, as it affects, on average, 12% of the global population during the productive years of work (30–45 years). During the period of exacerbation, patients’ capacity to work and their social activity are compromised, imposing a significant individual and economic burden worldwide [4,11,18,19].

Epidemiological data show that women have a superior incidence of this neurological condition (from the onset of puberty and throughout life), and a higher recurrence rate and duration of attacks than men. These sex-related differences have clinical relevance and should be considered during experimental studies. Nevertheless, it is crucial to note that this disorder affects both genders, underscoring the importance of studying and comprehending it in detail [20].

Migraine attacks are characterized by brain episodes that can occur over hours to days and are classically composed of four phases: premonitory, aura, migraine headache itself and postdrome [4,17,21,22]. The premonitory phase begins a few hours to days before the headache phase and is characterized by several symptoms, which include impaired concentration, fatigue, and neck stiffness. At the same time of the premonitory and migraine headache phases, around a third of patients experience the aura phase, which is characterized by symptoms related to visual, sensory, and/or motor disturbances. However, most patients do not experience this phase and have symptoms of nausea and sensitivity to light and sound associated with the headache [17,21]. After the headache phase, the postdrome phase is characterized by non-headache symptoms, which include fatigue, difficulty in concentrating, and neck stiffness, lasting up to 24–48 h [4,21]. Table 1 depicts the severity of the various phases of migraine and their associated symptoms.

### 2.1. Functional Anatomy and Pathophysiology

Although the pathophysiology and neurobiology of migraine are not totally understood, the investigations in this area have been evolving in recent decades [18,25]. In particular, there are several hypotheses that attempt to explain the underlying mechanisms of migraine in general. Initially, the vascular theory dominated but, currently, the development of migraine is suggested as a consequence of multiple primary neuronal impairments that cause alterations at intra- and extracranial level [4,25,26]. According to the most recent theory, migraine is a neurovascular disease, mostly associated with the activation of trigeminal nociceptors and with the secretion of inflammatory mediators that target the signaling pathways associated with central pain [18].

Based on the available clinical and imaging data, the hypothalamus plays a crucial role in the onset of a migraine attack and in the regulation of the intensity of the attack [18]. Anatomically, the hypothalamus has connections to the trigeminovascular system, the thalamus and brainstem neurons, which influence the autonomic and nociceptive regulation of this neurological condition [2]. More specifically, recent studies indicate that following hypothalamic activation, the trigeminal ganglion is activated and a release of calcitonin gene-related peptide (CGRP) occurs [26]. CGRP has a potent vasodilatory capacity and is mainly expressed in central and peripheral neurons [2,27]. Upon the release of CGRP by antidromic stimulation of trigeminal ganglion, it is possible to observe vasodilation of arteries in sensory organs, middle meningeal vasodilation (origin of the vascular theory), and mast cell degranulation (in rats), which can explain the main phenotypical alterations observed during an attack. Furthermore, the higher incidence of migraine attacks in women in comparison to men can be explained by hormonal factors, since the female sex hormone estradiol promotes the CGRP release in the trigeminal nucleus caudalis and in the trigeminal afferents in humans [20,28]. However, the release of CGRP cannot directly explain migraine pain, since not all vasodilators cause migraine pain [26]. In general, the transmission of pain is related with the activation of the receptors in the primary afferent fibers, namely the unmyelinated C-fibers and the myelinated Aδ-fibers. When a potential noxious stimulus occurs, both of these nociceptors are activated, and for this reason, the origin of the migraine pain signal also seems to be related with the sensitization of these two major classes of nerve fibers. More specifically, the release of CGRP from unmyelinated C-fibers TG neurons directly activates the calcitonin receptor-like receptor (CRLR), promoting an intracellular increase of cyclic adenosine monophosphate (cAMP). As a result of this increase, there is hyperexcitability of the Aδ-fibers, which transmit noxious information, leading to normal stimuli being felt as pain [26,29,30].

In addition to CGRP, stimulation of the Aδ- and C-fibers of the trigeminal nerve leads to the secretion of other biologically active substances that are known to promote neurogenic inflammation, namely, pituitary adenylate cyclase activating polypeptide (PACAP) and substance P. In particular, substance P has particular biological activities related to the vasodilatory and algogenic effect, the increase in vascular permeability, and the increased sensitivity to pain that come from its pro-inflammatory effects. PACAP is detected in high concentration in plasma during migraine attacks, and the effects of this polypeptide are related to vasodilation and perivascular inflammation derived from stimulation of C-fibers and degranulation of mast cells [18,25,31]. A study developed by Zagami et al. showed that, during a migraine attack, patients with moderate pain had PACAP levels of 36 ± 3 pmol/L, in contrast to patients without pain who had PACAP levels of 21 ± 3 pmol/L [32].

The midbrain periaqueductal gray contains longitudinally oriented neuronal columns that control nociceptive responses and allow the inhibition of the painful stimulus, when activated. However, second-order neurons (nerve cells that receive direct synaptic input from the first-order neurons), located in the trigeminal nucleus caudalis and upper cervical spinal cord, also contact with different sites in the brainstem, namely the periaqueductal gray, recognized for modulating noxious inputs, perturbing the neurons in that region and inducing migraine-like headache [4,18,33,34,35,36]. In addition, the serotonergic system (5-hydroxytryptamine/serotonin, 5-HT) from the brainstem raphe nucleus also appears to contribute convincingly to the pathophysiology of migraine. During migraine attacks, alterations in 5-HT metabolism and in central responses mediated by 5-HT are observed, suggesting that this neurological condition results from a central neurochemical disequilibrium arising from a low serotoninergic disposition. Indeed, although the full cascade of events is not yet fully understood, recent studies indicate that low serotonergic disposition promotes activation of the trigeminovascular nociceptive pathway [17,37]. Beyond headache, the complexity of symptoms that arise during the migraine phase appears to be related to connections between the hypothalamus and the thalamus. During this phase, the second-order trigeminovascular neurons send information to the thalamus, projecting to cortical regions related to sensory, cognitive and emotional functions, and which lead to the symptoms of allodynia, osmo-, photo- and phonophobia [2,18].

**Figure 1:**
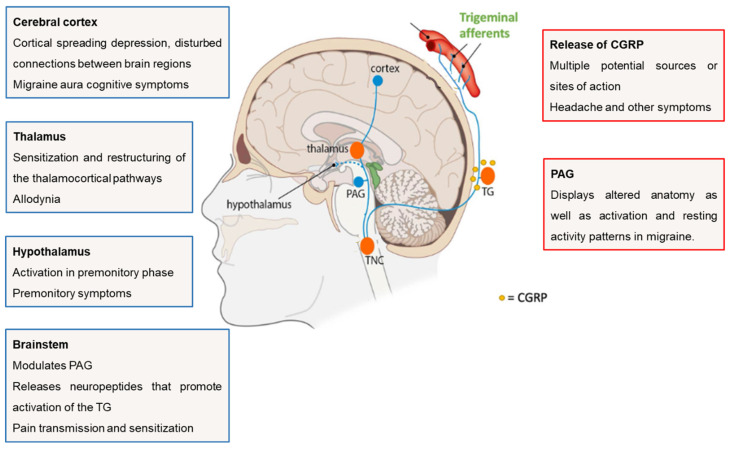
Activation of the trigeminovascular system during a migraine attack and the main brain regions involved in the mechanisms underlying this neurological disorder. Trigeminal ganglion (TG); trigeminal nucleus caudalis (TNC); calcitonin gene related peptide (CGRP); and periaqueductal gray (PAG). Adapted with permission from [28].

The involvement of the cerebral cortex in the genesis of migraine has also been studied over the last few years and, in fact, various alterations in the structure and function of cortical areas that are related to pain processing have been described. In particular, the involvement of the occipital cortex may explain the multiplicity of visual symptoms that patients experience, such as photophobia and scintillating scotoma [2]. Figure 1 outlines the activation of the trigeminovascular system during a migraine attack and the principal brain regions involved in the mechanisms underlying this neurological disorder.

Overall, migraine involves multiple elements of the central and peripheral nervous system—the hypothalamus, the brainstem, the thalamus, the cortex, and the trigeminovascular system—that renders migraine to be considered a complex and not fully understood medical condition. Nevertheless, because it is a common condition, there is an urgent imperative to further understand the neurobiology of the disease, which will prompt the discovery of novel treatment approaches to universally and effectively abort or prevent migraine attacks [25,33,38].

### 2.2. Pharmacological Treatment of Migraine

In the last two decades, breakthroughs in migraine pathophysiology have allowed the development of novel target-based therapeutic approaches and the elucidation of the pharmacotherapeutic approaches that have been used to treat migraine attacks [21]. Thus, recent years represent an especially exciting period in the area of treatment of this disorder as a result of the approval of several medications in the clinical field, particularly challenging the division of pharmacological treatments between acute and preventive treatments [2,5,39,40,41,42].

Both treatments can be subdivided into different lines of medication and should be used in a stepped care approach. The selection of the most suitable medication for each patient should be based on the patient’s clinical conditions and associated comorbidities, since each drug option has its own set of AEs and can be contraindicated for other medical conditions that migraineurs may also suffer [5,21]. Table 2 summarizes the main classes of drugs used in acute and preventive treatments of migraine, as well as their AEs and contraindications.

#### 2.2.1. Acute Treatment

The acute treatment of migraine aims primarily to decrease the duration and severity of an attack in order to restore the patient’s functional capacity [21]. This therapy is suitable for quick treatment, normally with a large single dose [17]. It is classified in a stratified manner, starting with first-line medications, followed by second-line, third-line and adjunct medications (Table 2) [5].

First-line medication consists in the use of non-steroidal anti-inflammatory drugs (NSAIDs), which is considered a nonspecific pharmacological treatment, although studies support their ability to reduce neurogenic inflammation and to promote the reversion of central sensitization associated with migraine attacks. In case of contraindication of NSAIDs, paracetamol can be used to reduce the severity of symptoms [5,21,25,41]. Antiemetic drugs can also be used as a first line of medication to decrease nausea and vomiting [21,25].

Patients for whom first-line medication does not provide adequate relief of migraine symptoms should turn to second-line medication that includes the triptans, which have well-documented effectiveness when taken early in a migraine attack [5,21,44]. Triptans are full agonists of presynaptic serotonin receptors 5-HT_1B/1D_, which inhibit CGRP release and, consequently, the neuronal excitability of Aδ-fibers. Furthermore, they inhibit the trigeminal nerve’s activation, preventing the release of vasoactive neuropeptides, and also promote vasoconstriction of the cranial arteries, which dilate during an attack, causing pain [2,26,41,42,43]. This class of drugs is, therefore, considered as specific antimigraine drugs and therapy is often substituted, as some patients may not present relief from symptoms with one triptan, but present relief with another [2,5,17].

Ergotamine and dihydroergotamine can also be used in the acute treatment of migraine, although they are not recommended because of their AEs. These ergot derivatives have a mechanism of action similar to triptans, activating 5-HT serotonergic receptors and, consequently, reducing trigeminal neuron activation. [21,25].

In case of contraindication or unsuccessful relieve of symptoms with the previously mentioned lines of medication, there is a third-line medication, which includes the ditans—a group of selective 5-HT_1F_ receptor agonists—and the gepants—a group of CGRP receptor antagonists [2,3,5,41,42].

Noteworthy, these drugs show AEs and contraindications (Table 2), which limit their use in the acute treatment of migraine, since it is recommended that patients do not use acute drugs frequently and repeatedly, mainly due to the risk of developing headaches [2,3,5,21,43,48].

#### 2.2.2. Preventive Treatment

In patients whose migraine attacks continues to affect quality of life, despite an optimized acute treatment, further preventive treatment must be considered. This type of therapy is indicated for patients suffering from migraine at least 4 days per month and aims to decrease the frequency and intensity of acute attacks. In addition, another indication for the use of preventive treatment is related to the overuse of acute medication [5,21,25,49].

Compared to acute medication, preventive treatment can also be classified as first-line, second-line, and third-line options (Table 2). In general, the selection of preventive medication (first-, second- and third-line drugs) is governed by clinical judgment of the individual case and depends on local practice guidelines and local availability [2,5,50]. First-line drugs include beta-blockers (metoprolol and propranolol) and an anticonvulsant (topiramate); second-line drugs include an antidepressant (amitriptyline), a calcium channel blocker (flunarizine) and an anticonvulsant (valproic acid); and third-line drugs include peptide monoclonal antibodies (mAbs) related to the calcitonin gene (erenumab, fremanezumab, and galcanezumab) [5,25,41,50].

In clinical use, and accordingly to the treatment guidelines, mAbs against the CGRP have been widely introduced given its fast onset of efficacy and mild AEs. However, many patients are forced to stop treatment with mAbs due to regulatory restrictions that limit their use, treatment-emergent AEs, and high cost [2,51,52,53,54]. Preventive treatment is often associated with poor patient compliance, as efficacy is rarely observed [2,5,45,46,47,55].

#### 2.2.3. Non-Invasive Strategies to Overcome Treatment Limitations

The treatment of migraine is hampered by several difficulties already mentioned in Section 2.2.1 and Section 2.2.2, which include insufficient headache relief, contraindications, AEs, and risk of developing headaches due to overuse of acute drugs [48]. Although it is estimated that approximately 90% of individuals use drugs for the acute treatment of migraine, about 36% of these patients interrupt the treatment as a result of these difficulties [7]. One of the main reasons for the lack of effectiveness of migraine treatment is the fact that 90% of the prescribed therapies are based on oral tablets. Although oral drugs offer advantages, mainly due to their easy and non-invasive administration, they can show slow absorption and onset of therapeutic effect, as well as extensive first-pass metabolism, which necessitates the administration of high doses, leading to high systemic levels and consequently the occurrence of AEs [6,7,8,9,56]. Oral administration can also be associated with inter- and intra-patient variability in drug absorption. Furthermore, patients often have gastrointestinal symptoms associated with migraine, namely, vomiting, delayed gastric emptying, and nausea, which can lead to the loss of the administered drug and to uncertainty about whether to re-dose [7].

In addition, after oral administration, there is another important limitation in the transport of the drug to the brain related to the BBB, which constitutes a physical barrier that isolates the CNS from the systemic circulation and limits the entry into the brain of more than 90% of the drugs developed to treat neurological diseases [57,58]. Beyond this barrier, the brain is also protected from the entrance of external materials by the cerebrospinal fluid (CSF). Nonetheless, when oral or intravenous drugs are used, researchers actually consider the BBB as the most limiting barrier for drug entry into the brain [59].

The BBB has an endothelial structure consisting of different molecular and cellular components, which includes tightly connected endothelial capillary cells, neurons, microglia, pericytes, astrocytes-end-feet, and extracellular matrices. Under normal conditions, the BBB’s main function is to maintain regular CNS activities, providing nutrients and protecting it from harmful substances that circulate in the bloodstream. Only lipophilic and low molecular weight molecules can easily cross the BBB endothelial cells [60]. However, most drugs developed for the treatment of CNS disorders do not have high lipid solubility and/or molecular mass greater than 400 Daltons, requirements for efficiently surpass the BBB. Thus, strategies need to be found to overcome and disrupt the BBB and potentiate the transport of therapeutic molecules into the brain. These strategies can be divided into two main categories: invasive and non-invasive strategies [12,57,60].

Intrathecal administration of drugs, convention-enhanced delivery, and use of implants/microchips are examples of invasive approaches. These techniques show disadvantages that are related to maintenance costs, the need to monitor the patient, and the risk of brain complications. Given these disadvantages, the use of non-invasive strategies emerges as an alternative approach for the delivery of drugs to the brain. Within these strategies, nose-to-brain delivery and nanocarriers-based drug delivery to the brain have been extensively studied in the last decades [12,60,61].

The nose-to-brain delivery (or IN delivery) is the only direct pathway to the brain where drugs do not need to cross the BBB. This route takes benefit from the linking between the olfactory and trigeminal nerves, located in the nasal cavity, and the brain to increase drug levels in the CNS and to decrease systemic side effects [14,60,62]. An overview of this route and of the mechanisms of drug transport to the brain are described in Section 3. The use of nanosystems via the nose-to-brain route has shown good results in promoting the accumulation of drugs in the brain and overcoming some of the limitations of this route [14].

### 2.3. Nasal Products Approved for the Treatment of Acute Migraine

Migraine patients experience recurrent symptoms of nausea, vomiting and gastroparesis, which effectively reduce the absorption and efficacy of orally administered drugs during a migraine attack [63]. Evidence shows that the IN route is particularly interesting as an alternative to the oral route for administering drugs to treat this neurological condition [9]. Thereby, currently, there are several IN products approved for the acute treatment of migraine (Table 3) [7,64].

In 1997, IMITREX^®^ and MIGRANAL^®^ were the first products approved in the USA as nasal sprays for the acute treatment of migraine with or without aura. The first product contains sumatriptan and the second contains dihydroergotamine mesylate—both are selective agonists of the 5-HT_1D_ receptor that mediates vasoconstriction of cranial blood vessels [66,67]. During the trials for IMITREX^®^ approval, pharmacokinetic studies demonstrated a maximum plasma concentration (C_max_) of 69.5 ng/mL and 12.9 ng/mL following subcutaneous and nasal administration of sumatriptan, respectively, and a higher bioavailability of the nasal spray compared to the subcutaneous route [65]. Simultaneously, pharmacokinetic studies developed for MIGRANAL^®^ demonstrated that the mean bioavailability following nasal administration was 32% compared to intravenous administration [67,68]. In 2003, a nasal spray containing zolmitriptan was approved by the Food and Drug Administration (FDA) under the commercial name of ZOMIG^®^. This product consists of a liquid formulation intended for the delivery of zolmitriptan to the nasopharynx and respiratory region. The pharmacokinetic studies conducted with this product demonstrated greater mean bioavailability following nasal administration of ZOMIG^®^ compared to an oral tablet [69]. In addition, a clinical study conducted in adults revealed a significantly higher percentage of migraineurs who achieved headache relief and a consistent pain response during treatment of multiple attacks post-treatment with ZOMIG^®^ vs. placebo [70,71].

In 2016, the FDA approved the ONZETRA^TM^ Xsail^TM^ nasal product, which consists of a breath-powered technology included in a single-use nosepiece containing sumatriptan to treat acute migraine. ONZETRA^TM^ Xsail^TM^ has been developed taking into account the physiology and anatomy of the nasal cavity [72]. Thus, in contrast to products developed so far, nasal administration of ONZETRA^TM^ Xsail^TM^ allowed the delivery of sumatriptan to the upper part of the nasal cavity, avoiding its deposition in the oropharynx or lungs. A study that compared the pharmacokinetics of 20 mg of IMITREX^®^ and 22 mg of ONZETRA^TM^ Xsail^TM^ showed that the breath-powered technology of ONZETRA^TM^ Xsail^TM^ led to 27% greater C_max_ (20.8 vs. 16.4 ng/mL) and 75% greater early exposure (AUC_0–15min_, 2.1 vs. 1.2 ng*h/mL), compared to IMITREX^®^ spray [73]. In 2019, the FDA approved an innovative product indicated for the treatment of migraine with or without aura—TOSYMRA^TM^. This innovation is due to the fact that this product contains sumatriptan and a permeation enhancer (0.2% 1-O-n-Dodecyl-β-D-maltopyranoside) that improves the absorption and bioavailability of the IN drug [75]. The studies carried out for this product demonstrated the mean bioavailability after nasal administration of TOSYMRA^TM^ 10 mg is 58%, when compared to 6 mg subcutaneous injection of the same drug [76]. In addition, pharmacokinetic studies comparing the administration of 10 mg of TOSYMRA^TM^ to 20 mg of IMITREX^®^ obtained C_max_ of 63.9 and 21.4 ng/mL and AUC_0–2h_ of 48.4 and 24.7 ng*h/mL for TOSYMRA^TM^ and IMITREX^®^, respectively, demonstrating that the drug administered in the TOSYMRA^TM^ was more rapidly absorbed [77]. In terms of safety and tolerability, a clinical study carried out in patients suffering from 2 to 6 migraine per month demonstrated that TOSYMRA^TM^ was well tolerated when used during 6 months [76].

In 2021, FDA approved Trudhesa^TM^ nasal spray. Trudhesa^TM^ is an ergotamine derivative, such as the nasal spray MIGRANAL^®^, which uses Precision Olfactory Delivery (POD^®^) technology for a rapid absorption and bioavailability of the drug. Trudhesa^TM^ is considered more effective than the nasal spray approved in 1997, since the product is delivered as a fine aerosol to the upper part of the nasal cavity and allows the administration of lower doses of dihydroergotamine mesylate [58,78].

In March 2023, the FDA approved Zavzpret™ which contains 10 mg of zavegepant—a calcitonin gene-related peptide (CGRP) receptor antagonist—in a single-dose nasal spray device. In phase 3 clinical trials, it was observed that 2 h following the first dose, a greater percentage of patients from the 10 mg zavegepant nasal spray group experienced less pain and unpleasant symptoms than patients from the placebo group. Additionally, a greater percentage of patients taking zavegepant compared to those taking placebo experienced pain relief at 15 min (15.9% vs. 8.0%), 30 min (30.5% vs. 20.3%), 1 h (43.3% vs. 37.3%) and 2 h (58.7% vs. 49.7%) and absence of pain between 2 and 48 h (12.4% vs. 8.7%), respectively. Indeed, all the products approved to date have shown, in phase 3 clinical trials, a significantly higher percentage of migraineurs with headache relief 2 h after treatment, with the exception of MIGRANAL^®^ and Trudhesa^TM^, which showed a higher percentage of migraineurs with headache relief only 4 h after treatment, compared to placebo. In addition, the pooled data from the phase 3 pivotal studies indicated that all the marketed products had a favorable safety profile. Despite this, around 2% of patients reported AEs for each of the products described [79].

## 3. Nose-to-Brain Route—An Overview

The nasal route has been extensively used for the local treatment of different diseases, administering corticosteroids, decongestants, and antihistamines. In the context of drug administration via the nasal cavity, the nose-to-brain route has been gaining increasing interest from the scientific community as an advantageous approach to improve the treatment of CNS disorders. This route is considered an alternative therapeutic approach to the intravenous and oral routes for administering drugs to the brain, as it avoids the need to surpass through the BBB and allows to obtain a high concentration of drug molecules in the cerebral region more quickly. This increased targeting of drugs to the brain offers important advantages, namely the use of smaller drug doses to achieve the same therapeutic effect and the reduction of systemic AEs [14,62]. This pathway also includes other advantages, such as the high blood flow and the large surface area for drug absorption, which allows the increase of drug bioavailability [57,59].

Thus, the nasal cavity has a set of anatomical characteristics advantageous for the administration of drugs, which also allows recognizing the IN route as a non-invasive route that allows a rapid onset of action and avoids hepatic first-pass metabolism and degradation of the drug in the gastrointestinal tract. In addition, the nose-to-brain route presents clinical advantages that increase patient acceptability, including the ease of carrying out non-painful self-administration, which is particularly important for migraine patients, where drug administration can occur during episodes of extreme nausea or vomiting [59,60]. Physiologically, the nose is responsible for breathing, smelling, regulating temperature, and removing external pathogens [57,80]. Anatomically, the nasal cavity has a total surface area of 160 cm^2^ and a length from the nostrils to the nasopharynx of 12 to 14 cm. The nasal septum divides this cavity in two identical parts, which are subdivided into three different regions: vestibular, olfactory, and respiratory [57,81].

### 3.1. Mechanisms of Drug Delivery to the Brain

**Figure 2:**
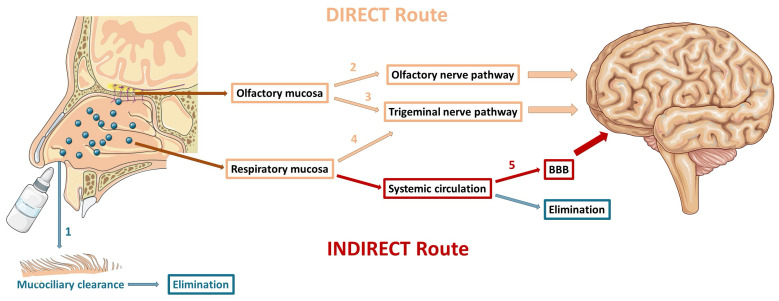
Overview of the different drug pathways after intranasal (IN) administration. (1) The drug is eliminated by the mucociliary clearance mechanism. DIRECT route: (2) olfactory nerve pathway—the drug is absorbed in the olfactory mucosa and passes to the brain through the olfactory nerves; (3) trigeminal nerve pathway—the drug is absorbed in the olfactory mucosa and passes to the brain through the trigeminal nerve; (4) trigeminal nerve pathway—the drug is absorbed in the respiratory mucosa and passes to the brain through the trigeminal nerve. INDIRECT route: (5) the drug is absorbed in the respiratory mucosa, reaches the systemic circulation and can surpass the blood-brain barrier (BBB) and reach the brain, or be eliminated before it reaches the brain. Adapted with permission from references [62,82].

The transport of drugs from the nasal cavity directly to the CNS occurs mainly through the olfactory and trigeminal nerve pathways. However, the drug can still reach the CNS indirectly via the systemic circulation. Figure 2 summarizes the different pathways that can be followed by drugs after IN administration.

The neuronal pathways of direct drug transport comprise the olfactory nerve pathway (via the olfactory mucosa) and the trigeminal nerve pathway (via the olfactory and respiratory mucosae). The olfactory nerve pathway allows to a faster transport of the drug to the brain (≈0.33 h), in relation to the trigeminal nerve pathway (≈1.7 h) and, therefore, seems to be more determinant in the direct transport of drugs from the nose to the CNS [57]. However, in addition to transport time, the physicochemical characteristics of the drug, the consistency of the formulation, and the type of administration device can also determine the route through which the drug transport occurs [62,81,83].

#### 3.1.1. Olfactory Nerve Pathway

In the olfactory mucosa, following IN administration drugs can pass to the brain via the olfactory nerves. Concisely, the drug passes along the olfactory nerves and through the olfactory bulb and, subsequently, reaches the brain area [57,60]. This transport can occur through intraneuronal or extraneuronal transport [14,57]. Intraneuronal transport consists of the endocytosis of the drug by the olfactory nerves, where the molecule is transported along the neurons (axonal transport) to the olfactory bulb and then is released into the brain by exocytosis. This is as a slow transport mechanism that requires hours or days for the molecules achieving the brain, due to the average axonal transport speed of 25 mm per day. However, some investigations have reported that the speed of transport depends on the characteristics of the transported molecule and on the diameter of the axon. Considering that the diameter of a human olfactory axon is about 100 to 700 nm, only molecules within this size range should be transported by intraneuronal transport [84,85]. Furthermore, the extraneuronal transport is faster (≈30 min) and drugs reach the brain through the gaps between the olfactory neurons. This transport can be subdivided in transcellular and paracellular transport. In the former, drugs cross the olfactory mucosa through the supporting cells; while in the latter, drugs cross the olfactory mucosa along the supporting cells. The latter occurs during the period in which olfactory neurons renew themselves, which is generally every 30–60 days. During renewal, there is an opening between the surrounding supporting cells of the epithelium, which allows drug access to the olfactory bulb and the brain. Notwithstanding, although it has been described that CNS neurons do not have the capacity for self-renewal and regeneration as they mature [86], it has recently been described that olfactory neurons have the potential to regenerate and renew themselves due to the ability of basal cells in the olfactory epithelium of the nasal cavity to function as pluripotent stem cells [87].

In general, lipophilic drugs follow transcellular transport via receptor-mediated endocytosis or passive diffusion; while hydrophilic and small drugs pass by paracellular transport through the tight junctions [57,58,64]. Noteworthy, although the transport of drugs from the olfactory region to the brain can occur inside or outside the nerves, it is very likely that drug transport combines different routes and not just a single route [57,58,60,88].

#### 3.1.2. Trigeminal Nerve Pathway

The trigeminal pathway involves drug transport via the trigeminal nerve, which is the largest cranial nerve and innervates both the respiratory and olfactory mucosae. The ophthalmic and maxillary branches are the most important for the delivery of drugs to the brain through this pathway, due to their direct connection with the brain [14,57,60]. As with the olfactory pathway, the physicochemical characteristics of the drug determine whether their transport occurs intracellularly, across axons, or extracellularly, through perineuronal channels, perivascular spaces, and lymphatic channels connected to the CSF and brain tissues. It has been reported that intracellular transport is a slow process and its contribution is not relevant to direct nose-to-brain transport in relation to the extracellular transport, which is faster [14,89]. Furthermore, it has been described that the direct nose-to-brain drug transport via the olfactory nerve pathway is greater than via the trigeminal nerve pathway. Indeed, the former occurs closer to the CSF, has less systemic exposure and the olfactory nerves are shorter than the trigeminal nerve [14,64].

#### 3.1.3. Indirect Transport

The indirect transport can occur after absorption of the drug in the respiratory mucosa and passage into the systemic circulation and represents a residual route in the transport of drugs to the brain regions after IN administration. Indeed, only molecules that are lipophilic and have a small molecular weight can easily pass from systemic circulation to the brain, through the BBB. In this way, the indirect route is less preferred, given the limitations offered by the BBB and peripheral AEs that can arise from the systemic distribution of the drug [14,81].

### 3.2. Challenges of Nose-to-Brain Drug Delivery

Although scientists have been working hard to study the physiology and anatomy of the nasal cavity, several questions remain to be answered regarding the exact transport pathway followed by drugs after IN administration and how they reach the brain [57,81]. Indeed, drug transport via the nose-to-brain route presents difficulties that must be considered during the development of IN formulations. These challenges are mostly related to the anatomical and physiological properties of the nasal cavity, the physiochemical characteristics of the drugs, and formulation-specific factors [12,14,81].

One of the main limitations of the IN route is the mucociliary clearance mechanism that is related to the presence of mucus associated with cilia movement, which reduce the retention time of the drug in the nasal mucosa and the consequent passage to the CNS. The mucociliary clearance is a defence mechanism of the respiratory tract against the entry of exogenous substances, which are retained in the mucus and eliminated through the combined movement of mucus and cilia, in a renewal process that takes place every 15–30 min [14,57,64,90]. Cilia act as a barrier to the penetration of particles from the outside and are present in the olfactory region in a non-motile form and in the respiratory region in a motile form. As it is expected, this natural defense mechanism affects the bioavailability of drugs that cannot remain long enough to penetrate the mucus and adhere to the nasal mucosa [59,84]. One of the key factors determining drug absorption is their lipophilicity, as drugs must have enough lipophilicity to permeate the biological membranes. In particular, particles larger than 1 µm in diameter and lipophilic molecules have more difficulty in penetrating the mucus layer compared to hydrophilic molecules, due to the high water content and the electrostatic, hydrophobic, and Van der Waals interactions with mucus [84,91,92]. Furthermore, the nasal mucosa contains enzymes, such as cytochrome P-450 isoforms, carboxylesterases, peptidases, and proteases, that can alter the solubility and chemical structure of IN molecules, changing their activity and permeation properties and, consequently, restricting their direct passage to the brain [12,81]. P-glycoprotein, a membrane transport protein found in the BBB, olfactory epithelium, and olfactory bulb, which acts by promoting the efflux of many molecules, can reduce the bioavailability of several drugs in the CNS [59,93]. Although the expression of the P-glycoprotein transporter in the olfactory epithelium is higher than in the respiratory epithelium, this is considered the area of greatest interest for the nose-to-brain drug transport. Indeed, this region lacks motile cilia that promote the mucociliary clearance mechanism and, therefore, the rate of turnover is slower, allowing an improved adhesion of the drugs to the nasal epithelium and, consequently, a greater absorption. However, targeting drugs to the olfactory region can be challenging, because this region has a small surface area and is located in the upper part of the nasal cavity. Furthermore, inappropriate administration of drugs intended for the olfactory nerve pathway can lead to their absorption in the respiratory region and subsequent passage into the systemic circulation, reducing the quantity of drug that directly reaches the brain and, consequently, increasing peripheral side effects. To circumvent this, the nasal delivery devices should deposit the formulation directly in the olfactory region of the nasal cavity [60,64,81]. However, patient-device interactions can hamper the accuracy of these devices, namely, positioning the device at an incorrect angle and inappropriate head position during administration have been described as the factors that most influence the deposition of the drug in the correct place within the nasal cavity [57]. Another limiting factor in targeting drugs to the brain following IN administration is the presence of tight junctions in the nasal epithelium. These protein complexes limit the passage of drugs towards the lamina propria and, thus, to the olfactory bulb and the brain. The study of tight junction permeability modelling has been crucial for the nose-to-brain route, maximizing drug passage to the lamina propria [64].

The physicochemical nature of the drug molecule has also a high impact on the absorption efficiency in the nasal mucosa and, therefore, on its bioavailability within the brain. More specifically, properties such as lipophilic/hydrophilic balance, molecular weight and degree of ionization (pKa) significantly limit the drugs that can be transported via this route and the efficiency with which they are absorbed through the nasal mucosa [64,81]. In general, drugs with molecular weight greater than 1 kDa tend to be stuck in mucus and rapidly eliminated; while drugs with molecular weight smaller than 300 Da are capable of sufficiently permeate the nasal epithelium and be transported directly to the brain, regardless of their physicochemical characteristics. In contrast, the direct transport of drugs with molecular weight greater than 300 Da and smaller than 1 kDa depends on their physicochemical properties, especially on their hydrophilic and lipophilic character and on the degree of ionization. Although hydrophilic drugs penetrate the mucus layer easier, investigations suggest that greater hydrophilicity and degree of ionization result in smaller nose-to-brain drug transport [12,60,64,81,92].

Factors related to the formulation are also essential for the efficacy of direct drug transport to the brain. In particular, the effectiveness of nasal absorption is affected by the pH of the formulation (which in turn affects the degree of ionization of the drugs), and by osmolality, viscosity, volume and pharmaceutical dosage form [94]. Formulations should have pH similar to that of the nasal mucosa, ranging between 5.5 and 6.5, to prevent infection avoid irritation and maintain the normal cilia movement, although this value can increase to 7.2–8.3, for example, in patients with rhinitis; the pKa of the drug and the amount of ionized and non-ionized drug molecules should be considered, as the nasal mucosa allows greater permeation of non-ionized drug molecules. In addition, the formulations should be isotonic so as not to interfere with the normal function of the nasal mucosa’s defense mechanisms; and have adequate viscosity, since although very viscous formulations promote contact with the nasal mucosa, they can decrease the diffusion of the drug and impair cilia movement [12,59,81,94,95].

A limiting condition related to the anatomy of the nasal cavity that should be considered is the amount of formulation that can be administered through this route. In fact, only a limited volume of formulation (100–200 μL) can be administered per nostril at a time. Several authors consider that this condition significantly restricts the usefulness of IN administration only to the most powerful drugs [13,81]. However, as the nose-to-brain route allows for a fast and direct transport of the drugs to the brain, the dose required can be significantly reduced and, therefore, the therapeutic results can be similar or even superior to those obtained using the other routes [58,81,96,97,98].

The safety of the formulations and drugs administered via IN is often questioned because they can cause toxic effects both in the nasal cavity and in the brain. Although safe and biocompatible excipients are required to compose pharmaceutical dosage forms, continuous IN formulations administration can lead to irritation and damage of the nasal mucosa and of the olfactory nervous system [58,99]. Therefore, chronic patients who require continuous administrations should be monitored [58,81].

Another impacting factor to be considered in relation to IN administration is the patient’s heterogeneity, since many patients have pre-existing illness (allergies or infections) that change the pH and enzyme composition of the nasal mucosa, influencing the interaction of the formulations with this mucosa and, consequently, drug absorption [83]. For example, migraine patients commonly experience autonomic dysfunction within the nose during an acute attack, which is associated with symptoms of congestion and rhinorrhea that alter the physiological conditions of the nasal mucosa [6,12]. In addition, different races and genders can also influence the IN administration, due to the anatomical and physiological differences among the distinct regions of the nasal cavity [100].

The above referred limitations suggest that it is important to perform in vivo studies, to better understand the function of the nasal cavity and to develop more specific formulations with greater efficacy and therapeutic safety [54]. Notwithstanding, the lack of in vivo studies in humans is limiting the clinical practice of this route. Indeed, rodents are the main models used in preclinical studies and are considered limited in the translatability of results to humans due to differences in anatomy, volume administered, and administration techniques [81].

More specifically, 50% of the nasal cavity of mice is covered by olfactory epithelium, while in humans this epithelium represents only 10% of the nasal cavity. Moreover, the maximum volume of formulation that can be administered in mice is smaller (20 to 30 μL), when compared to the volume administered in humans [81]. Furthermore, in the laboratory, when administering, it can be guaranteed that the animals are positioned at a correct angle for drug targeting, although the same cannot be ensured, for example, in patients with reduced mobility who need to self-administer. Finally, it is important to emphasize that the animals used in these studies are generally anaesthetized, which leads to a decrease in respiratory rate and drug clearance rate and, consequently, an increase in absorption, which is not the case in non-anaesthetized animals [64]. Several approaches have been studied to overcome the challenges of the nose-to-brain drug delivery (Table 4).

The strategies presented in Table 4 are essentially aimed at improving the absorption and transport of drugs from the nasal cavity to the brain, reducing the nasal and systemic toxicity of the formulations. Among these, those that circumvent the mucociliary clearance mechanism and nasal enzymatic activity are the most relevant, since these are the main limitations of IN administration [58,104].

Mucoadhesive agents, absorption enhancers and viscosity enhancers have been identified as the ones with potential to limit the mucociliary clearance mechanism and, therefore, to increase drug permeation through the nasal mucosa. Another method studied to increase the permeability of the nasal mucosa is the transient reduction of epithelial tight junctions. Several compounds have been used for this purpose, such as poly-L-arginine, papaverine, 12-O-tetradecanotlophorbol-13-acetate, and bisindolylmaleimide [64]. In addition, the absorption enhancers and chelating agents can also disrupt tight junctions and accelerate drug transport [81]. Thereby, the addition of these types of excipients should be considered fundamental during the formulation development process [58].

The main strategies that significantly increase the retention time of formulations in the nasal cavity include the use of hydrogels as formulation viscosity enhancers and nanoparticulate drug delivery systems as mucoadhesive systems [12,64]. Despite both proved to be effective, the use of nanoparticulate systems is considered unique, since they not only increase drug absorption, but also protect them from enzymatic degradation and efflux transport mechanisms mediated by the P-glycoprotein [60,64]. The use of enzyme modulators can also prevent drug degradation in the nasal cavity [60,64].

Another strategy described to improve the direct transport of drugs from the nose to the brain, involves reducing the drug delivery to the respiratory mucosa, avoiding an extensive systemic absorption. Thereby, one of the most effective strategies is the use of delivery devices that direct the formulation to the olfactory region [60,64].

Nasal devices include droppers, sprays, metered-dose spray pumps, squeeze bottles, needleless syringes, breath-powered bi-directional devices, pressurized metered-dose inhalers, pressurized olfactory delivery, nebulizers, and atomizers. Droppers and spray pumps are conventional methods of nasal administration [61]. Nevertheless, formulations administered by using these devices are less likely to reach the olfactory epithelium, due to their location in the upper part of the nasal cavity and the need for an appropriate head position at the time of administration. To overcome these disadvantages, it has been proposed the use of new devices that deliver drugs in different physical states (liquid or powder) [105]. Nonetheless, only the advanced nebulizer devices Precision Olfactory Delivery (POD^®^), breath-actuated bi-directional nasal device (Optinose^®^) and electronic atomizer device (ViaNase™) have been used in clinical studies for nose-to-brain delivery [106]. POD^®^ allows effective administration of both powder and liquid drug formulations to the olfactory region; as well as the Optinose^®^ that also aims to deliver liquid and powder formulations to the same region. In the latter device, the closure of the soft palate allows even less deposition of the formulation in the lower nasal regions and ensures that no flowing powder is deposited into the lungs. Less specific to the upper part of the nasal cavity, ViaNase^TM^ targets the formulation to the respiratory and olfactory regions [105,107]. Regarding the challenge related to the damage that formulations can cause to the nasal mucosa, the scientific community recommends the use of biocompatible, biodegradable and generally recognized as safe (GRAS) excipients when developing formulations [102]. Furthermore, to decrease irritation of the nasal mucosa it is recommended the use of buffers to keep the pH of the formulations similar to that of the nasal mucosa; humectants to keep the mucosa moist; and isotonic excipients to keep formulations isotonic and not alter the normal movement of the cilia [12,81,90].

Limitations associated to the lipophilic and hydrophilic characteristics of drugs have also led research for suitable strategies to overcome them. Generally, hydrophilic drugs have difficulty being absorbed by the nasal mucosa and transported to the brain via transcellular mechanisms. In contrast, the nasal mucosa has hydrophilic components and highly lipophilic drugs do not have the ability to cross the nasal epithelium and turn out to be eliminated. In the case of hydrophilic drugs, it is necessary to use methods such as the incorporation of absorption enhancers and/or nanoparticulate drug delivery systems is required; and for drugs with high lipophilicity, the prodrug approach can be used to design inactive hydrophilic moieties of active lipophilic drugs [12,58]. The encapsulation of lipophilic drugs in nanoparticulate delivery systems also improves the absorption of drugs in the olfactory region and, consequently, their accumulation in the brain [14].

Indeed, the incorporation of drugs in nanoparticles seems to resolve many of the limitations of the nose-to-brain route. In particular, in vivo animal studies have demonstrated that the use of nanoparticulate systems associated with mucoadhesive agents and/or absorption enhancers has shown an improved targeting of the drug to the brain [14,88]. Compared to other types of nanoparticles, lipid nanoparticles have greater advantages in terms of stability, manufacturing techniques, encapsulation efficiency of lipophilic drug molecules, scalability and drug targeting [108]. These features will be discussed in detail in the following sections.

To evaluate the different strategies described to overcome the limitations associated with the nose-to-brain route, is essential to conduct in vivo studies in humans. As mentioned, the number of these studies is limited and, therefore, the transfer of these formulations to clinical practice is limited. However, the need for new treatments has led scientists to respond to this limitation [83]. Recently, Sasaki et al. developed a combination system for nose-to-brain drug delivery that comprised a mucoadhesive powder formulation included in a specific nasal device for non-human primates (Fit-lizer™ Type A), as investigators evaluated the formulation in conscious cynomolgus monkeys (*Macaca fascicularis*). Non-human primates are models that present physical and genetic similarity to humans and, therefore, their use in preclinical investigations is an appropriate strategy to circumvent the insufficient number of in vivo studies in humans. In particular, the nasal anatomy and area of the olfactory region of the cynomolgus monkey is very similar to that of humans. The results of in vivo experiments using Manganese-Enhanced Magnetic Resonance Imaging (MEMRI) demonstrated the selective delivery of the formulation to the olfactory region. However, the contribution of the trigeminal pathway remained unclear and should be explored in future studies. Nevertheless, the results of this research represent an important step forward in evaluating the effectiveness of the nose-to-brain route and in in vivo studies that use nanoparticle systems for drug delivery through this route [103].

## 4. Main Features of SLN and NLC

As a result of their small size and unique properties, the use of nanoparticles as drug carriers has been the focus of interest of researchers worldwide, specially to improve treatments of different unmet medical needs [109]. These systems are able to protect and target drugs to specific sites, allowing for a reduction in peripheral toxicity and an increase in therapeutic benefits [110,111]. So far, different nanoparticulate carriers have been used for these purposes, such as lipid nanoparticles, polymeric nanoparticles, and inorganic nanoparticles, among others [112]. Recent studies have shown particular interest in lipid nanoparticles, as they show advantages over other types of nanoparticles, including biodegradability, biocompatibility, low toxicity, scale-up capacity, and prolonged drug delivery [111]. In general, lipid nanoparticles include liposomes and solid lipid matrix nanoparticles, specifically solid lipid nanoparticles (SLN) and nanostructured lipid carriers (NLC), though different nomination for lipid-based nanoparticles can be found elsewhere [109]. Nonetheless, although liposomes have been developed earlier and there are already some products approved for clinical use, liposome-based formulations show stability problems and the raw materials are expensive [113,114]. Therefore, SLN and NLC arise as a promising alternative, as they can overcome these limitations and have proven to be effective drug delivery systems. Their success can be demonstrated by the exponential increase in scientific publications on these systems [109,110]. In particular, publications from 2015 to date have increased from 682 to 1113 in PubMed (61.3%) and from 1141 to 2773 in Scopus (41.1%), which are remarkable numbers if we consider that the total period of publications on SLN is around 30 years, and on NLC around 20 years. SLN were the first generation of solid lipid-based carrier systems with sizes in the nanometer range and ability to encapsulate lipophilic drugs [115]. There are some references to the ability of SLN to encapsulate hydrophilic drugs of small molecular size, and even proteins and nucleic acids, although with less success [116,117,118,119]. In fact, the encapsulation of nucleic acids is more efficient with nanoparticles made up of cationic lipids, as seen recently with COVID-19 vaccines [120].

SLN formulations are aqueous dispersions of nanoparticles composed of 5 to 30% of one solid lipid and stabilized up to 5% by one/two emulsifier(s) [60,62]. Although SLN have advantages, the presence of a single lipid in their solid matrix can lead to stability problems during the storage period, due to polymorphic transitions that give rise to a more organized matrix and the consequent progressive expulsion of the drug and, generally, to the aggregation of the nanoparticles. To circumvent the challenges associated with the structure of SLN, researchers have developed a second generation of lipid matrix nanoparticles, which are the NLC [62,110,121].

NLC have a less organized solid matrix due to their lipid composition that contains a mixture of one solid with one liquid lipid, usually in a ratio of 70:30, respectively [14,111]. The addition of the liquid lipid to the nanoparticles solid matrix originates a different and disorganized inner structure that allows to incorporate a higher amount of drug, reduces drug expulsion and increases stability during storage [111]. In other words, NLC share the same advantages described for SLN and show even more advantages over the latter. The superior efficacy of NLC in relation to SLN has directed investigations towards the latter. However, investigations with SLN remain effective [60,122].

### 4.1. Specificities of SLN and NLC for Nose-to-Brain Drug Delivery

In vitro and in vivo studies regarding the use of SLN and NLC for nose-to-brain drug delivery have demonstrated superior efficacy in targeting drugs to the brain compared to the administration of non-encapsulated drugs or incorporated in another type of nanosystems [14,57,123,124,125]. Indeed, SLN and NLC show characteristics that allow to improve the treatment of brain disorders via the nose-to-brain pathway [16,60,126]. As detailed in Section 3.1, both intraneuronal and extraneuronal routes can facilitate direct transport to the brain via the olfactory and trigeminal nerves, likely through a combination of both mechanisms. Reports suggest that the olfactory nerve pathway is more efficient due to its faster transport rate compared to the trigeminal nerve. Consequently, given that olfactory axons have diameters ranging from 100 to 700 nm, only SLN or NLC within this size range are expected to be transported intact directly to the brain via intraneuronal transport. For extraneuronal transport, similar to lipophilic drugs, SLN and NLC are anticipated to utilize transcellular transport mechanisms, such as receptor-mediated endocytosis or passive diffusion. Noteworthy, to the best of our knowledge, there is no confirmed evidence that nanoparticles reach the brain intact, which is beneficial as it avoids local accumulation of excipients [14,57,58,60,64,84,85,88,90].

There are, however, some particularities in nose-to-brain transport that make it necessary to ensure certain characteristics of the formulations intended for the administration of drugs by this route, in order to guarantee their safe and effective use [14,57]. Among the requirements of nose-to-brain formulations, the particle size is one of the most important parameters to enable the transport of encapsulated drugs through olfactory neurons. The size of IN nanosystems should comprise a nanometric range of less than 200 nm. In addition, polydispersity index (PDI) values of less than 0.3 are desired to guarantee a monodisperse particle size distribution and a uniform drug absorption through the nasal mucosa. In general, a PDI value close to 0 means a monodisperse formulation and a PDI value close to 1 means a polydisperse formulation [57]. The surface charge of the nanoparticles is also an important parameter to take into consideration when designing the formulation. This parameter is given by the zeta potential (ZP) value and predicts the long-term physical stability of the formulation. In general, a ZP value close to ǀ30ǀ mV promotes repulsive forces between particles and prevents their aggregation, contributing to the stability of the nanocarriers. Researches indicate that positive ZP allows the nanoparticles to better interact with the mucin residues present in the nasal mucosa, which are negatively charged, and increases the residence time and adhesion of the nanoparticles to the nasal epithelium [12,57,84,127]. Additionally, the encapsulation efficiency (EE), which estimates the amount of drug encapsulated in the nanoparticles, must be greater than 80% [57,127,128].

To finalize the requirements of the nose-to-brain formulations, several studies state that IN lipid-based formulations should have viscosity, tonicity, solubility, permeability and pH adjusted to the physiological values of the nasal mucosa, avoiding discomfort and irritation after administration. Concerns related to these parameters have already been referred in Section 3.2 [62,81,90,128]. In addition, dose, sterility, and stability during storage are also relevant parameters during the development of nose-to-brain formulations [129]. Indeed, the nose-to-brain administration leads to a risk of bacterial infections in the brain, which is why the use of sterile nasal formulations is recommended. However, to date, there is no regulatory requirement for sterility and therefore non-sterile manufacture is an option for nasal sprays, as it represents a benefit from the point of view of the cost of manufacturing the formulations [130,131]. Concerning the stability of the formulations, this implies taking extra care with their transport and sometimes refrigeration during the storage period to ensure that the particle size, PDI, ZP, and EE remain stable [132,133,134,135].

The efficacy of the IN SLN and NLC formulations is also ensured by other specificities that the nanoparticles must present to overcome some of the challenges associated with the nose-to-brain pathway, namely, the mucociliary clearance mechanism, which reduces the contact time of the nanoparticles with the nasal mucosa and, consequently, the bioavailability of drugs in the CNS. Thus, an important particularity that nanoparticles should present to increase nasal retention time and drug permeability in the nasal mucosa is related to its adequate coating with molecules that show ability for mucoadhesion [60]. Usually, hydrophilic molecules show greatest ability to adhere to mucosal surfaces due to the formation of hydrogen bonds with the mucin of the nasal mucus. Examples of hydrophilic molecules commonly used as mucoadhesive agents are alginate, chitosan, and cellulose derivatives [60,136,137,138]. In this area, the results of in vivo studies have been very promising, indicating that SLN and NLC with mucoadhesive properties are an effective strategy for improving the residence time of drugs in nasal mucosa [139]. However, recent studies refer to the use of mucus-penetrating nanoparticles instead of mucoadhesive nanoparticles for this purpose. This approach suggests that improving the residence time of the formulation does not mean that the bioavailability of the drug is improved and, therefore, the nanoparticles must be able to penetrate the mucus, and not just adhere to the mucin in order to increase the contact time of the drug with the nasal mucosa [60]. Thus, researchers have been exploring polymers to produce mucus-penetrating nanoparticles. In particular, the coating with polyethylene glycol (PEG) molecules, an uncharged hydrophilic polymer that gives rise to neutral surface nanoparticles, allows the production of mucus-penetrating nanoparticles. Beyond PEG, the dextran-protamine complex can also be used for this purpose. This specific application of mucus-penetrating lipid nanoparticles in the development of IN formulations is still a recent area of research and will certainly be further explored in the future [60]. For the same purpose, although more explored, the use of biorecognition ligands on the surface of nanosystems also allows to improve their binding to the nasal mucosa in order to increase their absorption into the brain. In specific, proteins with receptors in the olfactory region, such as lectins, have been commonly used to coat the surface of nanoparticles and constitute a gold standard for drug targeting to the brain [14,59].

After developing IN formulations, researchers consider including them in spray devices specifically designed to nose-to-brain delivery that deposit droplets in the olfactory region. One of the parameters that has become increasingly important to evaluate after including formulations in nasal devices is the characterization of the spray to control the quality of the final product. Researches demonstrated that droplets produced by different nasal spray devices are usually too large or, in the case of suspensions, individual droplets may contain multiple nanoparticles with drug or none at all. Various methodologies have been investigated that are or have the potential to be used to assess the droplet/particle size distribution of nasal delivery products [140].

In the most recent guidance, the FDA requests for the measurement of droplet size distribution obtained by laser diffraction (LD) at all stages of spray development, and also measurements at the beginning and end of the unit’s lifetime, evaluating two different distances from the actuator orifice. In general, the LD technique is suggested as the method of choice for the determination of droplet size in vitro and as a required technique for routine quality control tests. However, given that currently regulatory authorities and industry look to establish a link between in vitro tests and in vivo outcomes, this technique is not the most appropriate for this purpose, since it does not provide aerodynamic data and does not allow the chemical identification of the imaged particles and thus drug cannot be differentiated from excipient and agglomerates [140,141]. In addition, this technique does not guarantee that the size distribution is representative of the product as it tends to overestimate the number of small droplets [142]. For these reasons, the particle size measurement methodology has included new techniques, in particular, the Morphology-Directed Raman Spectroscopy (MDRS) [143,144]. This technique combines automated imaging and Raman Spectroscopy that allows the identification of individual particles and the collection of Raman Spectra for each particle, providing information on the morphological characteristics of the particles (size and shape) and chemical properties. Thus, it is through the result of optimizing these parameters that differentiation between the drug and the excipients is possible [127]. Despite the usefulness of this technique, there is currently no specific FDA guidance on MDRS [143,145].

Another requisite of IN formulations is to achieve droplets deposition in the upper region of the nasal cavity. To this end, the researchers are carrying out in vitro studies on anatomical models of the nasal cavity, which will allow more detailed identification of the sites where the formulation is deposited after administration of the IN spray. [127,146,147,148]. Indeed, recent results from FDA-commissioned studies demonstrated that anatomically accurate nasal models, along with digital simulation, can be used to analyze regional deposition [149,150]. In particular, the idealized nasal inlet, the Alberta Idealized Nasal Inlet (AINI), which contains four distinct anatomical regions (vestibule, nasopharynx, olfactory, and turbinates), was developed to mimic in vivo deposition [151]. A study developed by Chen et al. compared, over a range of different actuation angles, the deposition of a nasal spray solution marketed in vitro with AINI using gamma scintigraphy. The results shown that the AINI represented well the average in vivo deposition in this range of drug products. In particular, the AINI predictions for deposition of solution were statistically comparable to the previously obtained in vivo results [151,152]. Cunha et al. developed in situ hydrogels with rivastigmine-loaded NLC and rivastigmine-loaded nanoemulsion and evaluated the deposition profile of the developed formulations using the same nasal cast (AINI). The formulations were previously placed in nasal sprays and the results of nasal deposition on the olfactory region demonstrated that the % of deposited drug was 7.57 ± 0.13%, and 7.63 ± 0.678% for in situ hydrogels of rivastigmine-loaded NLC and rivastigmine-loaded nanoemulsion, respectively; and 4.48 ± 0.18% and 3.91 ± 0.22% for rivastigmine-loaded NLC and rivastigmine-loaded nanoemulsion alone, respectively, revealing a 2-fold higher deposition of the in situ hydrogels in the olfactory region, which is fundamental for the occurrence of the nose-to-brain transport [125]. Recently, Costa et al. developed and in situ hydrogel containing diazepam-loaded NLC and evaluated its deposition pattern in a 3D-printed human nasal cavity model, which was previously developed from a computerized tomography scan of a patient with healthy nasal airway passages. The results of the deposition studies allowed to detect the best nasal deposition profile, namely, when the formulation was administered at an angle from horizontal plane of 75°, without airflow, which resulted in 47% of the administered dose being deposited in the olfactory region [153]. The promising results of these studies will lead to an increase in the use of anatomical nasal cavity models to characterize nasal drug products, although it is necessary to validate these in vitro methods with results from in vivo studies, which should be conducted in animals, preferably non-human primates, and clinical studies in humans with the final SLN or NLC formulations for IN administration, to confirm their safety and efficacy for clinical use [140,148]. On the other hand, in vitro biocompatibility studies in cell cultures and ex vivo studies in organs or tissues should also be performed before starting in vivo studies [57,128].

### 4.2. Recent In Vivo Studies with SLN and NLC to Improve the Treatment of Acute Migraine via the Nose-to-Brain Route

Regarding the potential of SLN and NLC to target drugs to the brain, increasing the speed of action while reducing systemic toxicity and the therapeutic dose, the scientific community has been investigating the use of these nanosystems in the treatment of acute migraine [126]. Indeed, IN administration of drugs encapsulated in lipid nanoparticles used for the treatment of neurological diseases has shown promising results in vivo, compared to administration by other routes and/or administration of the drugs in solution or suspension [60]. These studies generally precede clinical trials and provide information on the pharmacodynamic, pharmacokinetic and absorption profiles of drugs in the human nasal cavity [13,57]. In particular, pharmacokinetic studies allow to evaluate the toxicity and therapeutic activity of the drug in the brain through different parameters, such as the maximum drug concentration (C_max_), the time required to reach the maximum drug concentration (T_max_); the area under the curve (AUC); the mean retention time of the formulation in the brain and the brain/blood ratio (i.e., the bioavailability of the drug in the brain). Moreover, with the values of these parameters it is possible to calculate two important neuropharmacokinetic parameters important, namely, the DTE (drug targeting efficiency), which represents the accumulation of drug in the brain after IN administration vs. intravenous administration (or other parenteral administration), and the DTP (drug targeting potential), which represents the amount of drug that reaches the brain via direct transport (i.e., via the olfactory and/or trigeminal pathways) [14,84,154,155]. When the DTE values are greater than 100, this means that the drug was directed to the brain more effectively through IN administration vs. intravenous administration; and when the DTP values are greater than 0, this means that targeting occurred mainly through the olfactory and/or trigeminal pathways. DTP values equal to 100 mean that the drug was absorbed in its totality by the direct route, following IN administration [156].

The following paragraphs and Table 5 summarize the pharmacokinetic results of the most relevant studies with drug-loaded SLN and NLC, and the respective drug in solution, administered intranasally and intravenously, for the treatment of acute migraine. In these studies, pharmacokinetic results with oral tablets are also shown for possible comparison of the values of AUC, T_max_ and C_max_.

Youssef et al. developed SLN loaded with almotriptan malate (ALM) dispersed in an in situ nasal mucoadhesive gel. From in vivo studies in Female Sprague Dawley rats, pharmacokinetic and biodistribution evaluation demonstrated that IN ALM-loaded SLN in in-situ gel allowed a rapid delivery of ALM to the brain (T_max,brain_ = 0.17 h) in comparison with IN free ALM in-situ gel (T_max,brain_ = 2 h) and IV ALM solution (T_max,brain_ = 0.5 h). The studies revealed higher maximum brain concentration (2.41 ± 0.04 µg/mL) and lower plasma concentration (2.69 ± 0.02 µg/mL) for IN ALM-loaded SLN in-situ gel (versus IN free ALM in-situ gel and IV ALM solution, with a brain concentration of 1.43 ± 0.02 µg/mL and 1.23 ± 0.02 µg/mL and plasma concentration of 3.09 ± 0.05 µg/mL and 3.20 ± 0.06 µg/mL, respectively), showing the notable superiority of the SLN formulation for the targeting of the drug to the brain, and simultaneously, its ability to reduce drug systemic exposure. The neuropharmacokinetic parameters also confirmed these evidences, revealing higher DTE and DTP values (335.7% and 70.21%) for the ALM-loaded SLN in-situ gel, compared to free drug in-situ gel (255.1% and 60.80%), respectively, which means a 1.3-fold increase in DTE. Furthermore, toxicological results confirmed the safety profile of in-situ gel based ALM-loaded SLN for IN administration through the evaluation of biomarkers and histopathological examination [157]. In another study, Salem et al. developed IN ALM-loaded NLC and performed in vivo studies, namely in albino male rabbits, with the aim of comparing the pharmacokinetic parameters of the optimized formulation with an IN ALM solution and oral market ALM product. Thus, after the conclusion of the studies, the results revealed a significantly higher C_max,brain_ value for the optimized IN ALM-loaded NLC (3.44 µg/mL) compared to those of the IN ALM solution (0.48 µg/mL) and oral market ALM tablet (0.52 µg/mL). ALM concentration reached its maximum value in brain after 0.17 h, 0.33 h, and 1 h following administration of IN ALM-loaded NLC, IN ALM solution and oral ALM tablet, respectively. In addition, the AUC_0–8h_ value obtained in the brain was also higher for IN ALM-loaded NLC (27,291.00 µg*h/mL) compared to IN ALM solution (3387.00 µg*h/mL) and oral ALM tablet (5982.60 µg*h/mL), indicating that nose-to-brain delivery of ALM-loaded NLC may represent a potential platform for the effective treatment of migraine [158].

Tripathi et al. investigated the success of an IN cinnarizine (CIN)-loaded NLC in situ gel and compared the results with an IN CIN solution. The pharmacokinetic studies in Male Wistar rats revealed an approximately two-fold increase in the concentration of CIN in the brain with the CIN-loaded NLC in situ gel (786.65 ± 7.4 μg/mL), when compared with CIN solution (380.73 ± 2.41 μg/mL). Thus, the results indicated that IN administration of CIN-loaded NLC in situ gel could be a step forward in the development of safe, effective, and improved drug delivery to the brain [159].

Bakshi et al. developed rizatriptan-loaded SLN and tested its efficacy for brain delivery after IN administration, compared with the IV administration of the same formulation and with a commercially available oral formulation of rizatriptan. The in vivo studies in Wistar rats (aged 4–5 months) of both sexes showed that when administered IN the maximum brain concentration of the SLN formulation (583.20 µg/mL) was higher than that achieved when administered IV (351.29 µg/mL) and higher than the concentration obtained for the marketed oral formulation (79.84 µg/mL). Furthermore, T_max_ values showed that the maximum brain concentration was obtained in less time (1 h) when the formulation was administered IN, twice longer when the formulation was administered IV (2 h), and nearly four times longer (4 h) to absorb when the drug was administered via the oral route, which shows a preferential transport of the formulation through the nose-brain pathway. However, the DTE (50.52%) and DTP (−97.88%) values do not indicate a more effective drug brain targeting after IN administration versus IV administration, and this is due to the C_max,blood_ value that was higher for IN administration (955.18 µg/mL and 175.12 µg/mL, respectively). In addition, the DTP value indicates that after IN administration most of the drug was absorbed via the indirect route [160]. In contrast, Masjedi et al. demonstrated that IN administration of sumatriptan-loaded NLC is more effective in targeting the drug to the brain, compared to IV administration of sumatriptan-loaded NLC and IN and IV administration of drug solutions. This fact was demonstrated by the results of the pharmacokinetic studies performed in male Sprague Dawley rats, which showed a C_max,brain_ value of IN sumatriptan-loaded NLC of 5.6, 7.4 and 9.4-fold higher than that obtained for IN sumatriptan solution, IV sumatriptan solution, and IV sumatriptan-loaded NLC, respectively. Furthermore, the AUC_0–4h_ brain value of the IN sumatriptan-loaded NLC was 12.95- and 7.70-fold higher than those of the IV sumatriptan solution, and IV sumatriptan-loaded NLC, respectively. Regarding the values of DTE (258.02%) and DTP (61.23%), these indicated higher partitioning values of the drug in the brain to plasma and suggest a greater suitability of the NLC for transporting the drug directly from the nose into the brain [161].

Kataria et al. developed IN zolmitriptan-loaded SLN and compared its in vivo efficacy in male Wistar rats with a marketed nasal zolmitriptan formulation (Zolmist nasal spray) and a zolmitriptan solution. The researchers evaluated various pharmacokinetic parameters, such as C_max_, T_max_ and AUC, although they did not have results for DTE and DTP since all the formulations were administered IN. The results showed that zolmitriptan-loaded SLN had a higher T_max_ value (0.5 h) than the other tested formulations, possibly due to a slower drug release pattern and the presence of Pluronic F-68 in the formulation that interacts longer with olfactory neurons. In this study, it was also confirmed that the encapsulation of drugs in SLN improves targeting of the drugs to the brain, in particular, the encapsulation of zolmitriptan in SLN originated a higher concentration of the drug in the brain (42.08 ± 1.32 ng/mL), when compared to the marketed formulation (32.34 ± 2.50 ng/mL) and drug solution (34.53 ± 1.56 ng/mL) [10].

## 5. Conclusions

From the results of the in vivo studies reported in this review, we can conclude that the nose-to-brain route is a promising alternative to conventional routes for improving direct drug transport to the brain in the treatment of acute migraine. Furthermore, the encapsulation of drugs in SLN or NLC is advantageous, since several studies have confirmed that these nanoparticles significantly improve the concentration of drugs in the brain after IN administration, compared to the IV and oral routes, and/or to the administration of free drugs.

The incorporation of SLN or NLC into in situ-forming hydrogels results in formulations with a longer residence time in the nasal cavity, which makes it possible to obtain a higher percentage of drug in the brain. However, there are particularities related to the characteristics of SLN and NLC that remain open for research. In particular, their ability to co-encapsulate drugs is considered a focus of interest for the treatment of acute migraine, since it has already been shown that the combined administration of NSAIDs and triptans can prolong the therapeutic effect and prevent further relapses. Currently, to the best of our knowledge there are no studies on the co-encapsulation of these two classes of drugs in SLN or NLC, which means that this is an open research field. Indeed, the area of co-encapsulation should continue to be a focus of interest among the scientific community, since its introduction into the clinical field could bring high benefits. To this end, it is important that, in the future, researchers include more reliable models for carrying out in vitro and in vivo studies, and ensure greater correlation between the studies and the clinical translation of the data obtained.

## Figures and Tables

**Table 1:** Different stages of migraine and associated symptoms. Adapted with permission from [23,24].

Phases of Migraine	Premonitory (Few Hours to Days)	Aura (5–60 min)	Headache (4–72 h)	Postdrome (24–48 h)
Associated symptoms	Impaired concentration	Numbness of face	Giddiness	Cognitive difficulties
	Mental slowing	Expressive language dysfunction	Insomnia	Lack of comprehension
	Speech dysfunction	Scintillating scotoma	Depressed mood	Depressed mood
	Drowsiness	Flashes of lights	Anxiety	Euphoric mood
	Yawning	Scotoma	Nasal congestion	Somnolence
	Fatigue	Paraesthesia/numbness	Neck pain/stiffness	Asthenia
	Food cravings	Motor dysfunction	HEADACHE Unilateral Severe disability Worsens with activity Throbbing	Tiredness
	Neck pain and stiffness		ASSOCIATED SYMPTOMS Nausea and vomiting Photophobia/phonophobia	Diuresis
	Photophobia			
	Nausea			
	Anorexia			
	Diarrhea			

**Table 2:** Different classes of drugs used in acute and preventive treatments, adverse effects, and contraindications.

Treatment	Drug Class	Drug	AEs	Contraindications	References
Acute	First-line medication				
	Non-steroidal anti-inflammatory drugs (NSAIDs)	Acetylsalicylic acid	Gastric effects	Patients with inflammatory bowel disease, renal dysfunction and who have had gastric bypass surgery.	[5,21]
		Ibuprofen			
		Naproxen			
		Diclofenac potassium			
	Other simple analgesics	Paracetamol	Gastrointestinal effects	Patients with hepatic disease and renal failure.	
	Antiemetic drugs	Metoclopramide	Drowsiness, weight gain, blurred vision, cardiac arrhythmias, urinary retention, extrapyramidal symptoms, and infertility	Patients with gastrointestinal bleeding, epilepsy, renal failure, cardiac arrhythmia, and Parkinson’s disease.	
		Chlorpromazine			
		Prochlorperazine			
	Second-line medication				
	Triptans	Sumatriptan	Nausea, dizziness, coronary vasoconstriction, chest pressure and tingling in the limbs	Patients with cardio- or cerebrovascular disease, uncontrolled hypertension, ischemic bowel, pregnant patients, or those who have used another triptan in the last 24 h.	[3,42,43,44]
		Zolmitriptan			
		Rizatriptan			
		Naratriptan			
		Almotriptan			
		Frovatriptan			
	Third-line medication				
	Ditans	Lasmiditan	Dizziness, nausea and somnolence	Pregnant women and patients using drugs that are P-glycoprotein substrates.	[2,3,5,42]
	Gepants	Ubrogepant	Fatigue and nausea	Patients with hypersensitivity and hepatic impairment.	
		Rimegepant			
Preventive	First-line medication				
	Beta blockers	Metoprolol	Dizziness, cold hands or feet and difficulties in sleeping	Patients with asthma, cardiac failure, Raynaud disease, atrioventricular block and diabetes *mellitus*.	[5,41,45,46]
		Propranolol			
	Anticonvulsant	Topiramate	Fatigue, cognitive disturbance, weight loss and paresthesia	Pregnant and lactating patients; and patients with nephrolithiasis and glaucoma.	
	Second-line medication				
	Antidepressant	Amitriptyline	Dry mouth, fatigue, dizziness and sweating	Patients with age ˂ 6 years, glaucoma, prostatic adenoma hyperplasia and heart insufficiency.	[5,46]
	Calcium channel blocker	Flunarizine	Fatigue, weight gain, depression, hyperkinesia, tremor, parkinsonism and gastrointestinal side effects	Patients with familial parkinsonism, focal dystonia and depression.	
	Anticonvulsant	Valproic acid	Fatigue dizziness, tremorand elevation of liver enzymes/disturbance in liver function	Patients with liver failure, pregnancy, alcoholism and polycystic ovaries.	
	Third-line medication				
	Calcitonin gene-related peptide monoclonal antibodies	Erenumab	Constipation, gastric pain, and chest pain	Patients with inflammatory bowel disease, coronary heart disease, chronic obstructive pulmonary disease and subarachnoid hemorrhage.	[5,47]
		Fremanezumab			
		Galcanezumab			

AEs, adverse effects.

**Table 3:** Nasal products approved for the acute treatment of migraine.

Drug	Product Details	Brand Name	Key Results	References
Sumatriptan	Dose: 5, 10, or 20 mg Liquid formulation delivered via traditional nasal spray	IMITREX^®^	Pharmacokinetic studies demonstrated a C_max_ blood of 69.5 ng/mL and 12.9 ng/mL following subcutaneous and nasal administration of sumatriptan, respectively. Pharmacokinetic studies demonstrated that the mean bioavailability following nasal administration is 15.8%, compared with the subcutaneous route. Greater percentage of patients had headache relief 2 h after treatment with 10 or 20 mg of IMITREX^®^ vs. placebo. Frequent AEs include nasal cavity/sinuses discomfort, burning, dizziness, nausea, vomiting, unusual taste and throat discomfort.	[65,66]
Dihydroergotamine mesylate	Dose: 2 mg Liquid formulation delivered to the respiratory region	MIGRANAL^®^	Pharmacokinetic studies demonstrated that the mean bioavailability following nasal administration is 32%, compared with the intravenous administration. Greater percentage of patients had headache relief 4 h after treatment with 2 mg of MIGRANAL^®^ vs. placebo. Frequent AEs include rhinitis, nausea, unusual taste, application site reactions and dizziness.	[67,68]
Zolmitriptan	Dose: 2.5 or 5 mg Liquid formulation delivered to the nasopharynx and lower nasal space	ZOMIG^®^	Pharmacokinetic studies demonstrated that the mean bioavailability following nasal administration is 102%, compared with the oral tablet. Greater percentage of patients had headache relief 2 h after treatment with 2.5 or 5 mg of ZOMIG^®^ vs. placebo. One multi-attack trial for adults showed that the headache response with ZOMIG^®^ was consistently maintained during the 2 h. Frequent AE include unusual taste (adolescents), paresthesia, hyperesthesia and somnolence.	[69,70,71]
Sumatriptan	Dose: 22 mg Nasal powder delivered via breath to the upper nasal space	ONZETRA^TM^ Xsail^TM^	Pharmacokinetic studies demonstrated that administration of sumatriptan nasal powder (ONZETRA^TM^ Xsail^TM^) resulted in 27% higher C_max_ (20.8 vs. 16.4 ng/mL) and 75% higher early exposure (AUC_0–15min_, 2.1 vs. 1.2 ng*h/mL) comparative to the sumatriptan nasal spray (IMITREX^®^). Pharmacokinetic studies demonstrated that the mean bioavailability following nasal administration is 19%, compared with the subcutaneous route. Greater percentage of patients had headache relief 2 h after treatment with 22 mg ONZETRA^TM^ Xsail^TM^ vs. placebo. Frequent AEs include unusual taste, nasal discomfort and rhinorrhea.	[72,73,74]
Sumatriptan	Dose: 10 mg Liquid formulation containing a permeation-enhancing excipient (0.2% 1-O-n-Dodecyl-β-D-maltopyranoside)	TOSYMRA™	Pharmacokinetic studies comparing a single dose of 10 mg TOSYMRA^TM^ to 20 mg IMITREX^®^ demonstrated that TOSYMRA^TM^ was more rapidly absorbed, with C_max_ values of 63.9 and 21.4 ng/mL and AUC_0–2h_ values of 48.4 and 24.7 ng*h/mL for TOSYMRA^TM^ and IMITREX^®^, respectively. Pharmacokinetic studies demonstrated that the mean bioavailability following nasal administration is 58%, compared with the subcutaneous route. Greater percentage of patients had headache relief 2 h after treatment with 10 mg TOSYMRA^TM^ vs. placebo. Frequent AEs include application site pain and reaction, unusual taste, upper respiratory infection, sinusitis and nasopharyngitis.	[75,76,77]
Dihydroergotamine mesylate	Dose: 1.45 mg Liquid formulation delivered to the upper nasal space	Trudhesa^TM^	Greater percentage of patients had headache relief 4 h after treatment with 2 mg Trudhesa^TM^ vs. placebo. In patients with migraine-associated nausea, photophobia, and phonophobia at baseline there was a lower incidence of these symptoms at 2- and 4-h following administration of Trudhesa^TM^ nasal spray vs. placebo. Frequent AEs include application site reaction, rhinitis, nausea, vomiting, somnolence, pharyngitis and diarrhea.	[58,78]
Zavegepant	Dose: 10 mg Liquid formulation delivered via nasal spray	Zavzpret™	Greater percentage of patients had headache relief 2 h after treatment with 10 mg Zavzpret^TM^ vs. placebo. Frequent AEs include unusual taste, nausea, nasal discomfort, and vomiting.	[79]

AEs, adverse effects; C_max_, maximum drug concentration; AUC, area under the curve.

**Table 4:** Limitations and strategies to improve the nose-to-brain drug delivery.

Limitations	Strategies	Description	References
Mucociliary clearance mechanism	Increased contact time of the formulation with the nasal mucosa for improved absorption of the drug	Absorption enhancers: cyclodextrins, sodium hyaluronate, Cremophor RH40, chitosan and cyclopentyladenosin	[58,59,64]
		Mucoadhesive agents: chitosan, and carboxymethylcellulose	[12,58]
		Viscosity enhancers: pectin, Pluronic^®^, Carbopol^®^, cellulose derivatives and chitosan	[12]
		Mucoadhesive systems: nanoparticulate drug delivery systems	[12,14,59,60,64]
Enzymatic and P-glycoprotein activity	Disturb the normal function of enzymes in the nasal epithelium	Enzyme modulators: P-glycoprotein inhibitors and CYP450 inhibitors	[59,60,64]
	Protection of drugs against enzymatic degradation and efflux transport mechanisms	Nanoparticulate drug delivery systems	[60]
Systemic absorption	Prevent deposition of the formulation in the respiratory region	Delivery devices designed to deposit the formulation in the olfactory region: ViaNase™, SipNose, OptiMist™, Precision Olfactory Device (POD^®^), VersiDoser^®^, VRx2^TM^, DART^TM^ and MAD Nasal^TM^	[59,60,64]
Tight junctions	Transiently decrease nasal epithelial tight junctions’ tightness	Compounds that modulate the permeability of tight junctions: chitosan, 12-O-tetradecanotlophorbol-13-acetate (TPA), papaverine, poly-L-arginine and bisindolylmaleimide	[64]
		Chelating agents: disodium ethylenediaminetetraacetate (EDTA)	[81]
		Absorption enhancers: polysorbate 80, propylene glycol, and polyethylene glycol 400	[81]
Physicochemical characteristics of drug molecules	Increase the nasal permeability of hydrophilic drugs	Nanoparticulate systems	[58,59,101]
		Absorption enhancers: cyclodextrins and chitosan	
	Increase the nasal permeability of lipophilic drugs	Nanoparticulate drug delivery systems	[14]
		Prodrugs	[12]
Damage to the nasal mucosa	Appropriately select the excipients of the formulation	Excipients generally recognized as safe (GRAS)	[102]
	Keep nasal mucosa moist	Humectants: glycerin, sorbitol, and mannitol	[12]
	Formulations with pH similar to the nasal cavity (5.5–6.5)	pH adjustment and buffers: citric acid, sodium chloride, sodium hydroxide, hydrochloric acid, and potassium phosphate	[81]
	Isotonic formulations	Isotonizing excipients: glycerin, sodium chloride, glucose or dextrose	[90]
Insufficient in vivo studies in humans	Use non-human primates with anatomical and physiological resemblance to humans	Preclinical investigations with cynomolgus monkey (*Macaca fascicularis*)	[103]

**Table 5:** Relevant results of pharmacokinetic studies with different drugs encapsulated in SLN or NLC to improve the treatment of acute migraine.

Drug	Formulations Tested	Constituents of SLN and NLC	AUC_0-t_ brain ± SD AUC_0-t_ blood ± SD (µg*h/mL)	T_max_ Brain (h)	C_max_ brain± SD C_max_ blood ± SD (µg/mL)	DTE (%)	DTP (%)	Relevant Results	References
Almotriptan malate (ALM)	IN ALM-loaded SLN in-situ gel	Solid lipid: Precirol^®^ ATO 5 Emulsifier(s): Polyvinyl alcohol (PVA) and Poloxamer 188	7.87 ± 0.09 8.77 ± 0.08	0.17	2.41 ± 0.04 2.69 ± 0.02	335.7	70.21	Higher C_max_ brain of IN ALM-loaded SLN in-situ gel (1.7-fold vs. IN free ALM in-situ gel and 2-fold vs. IV ALM solution); Faster onset of IN ALM-loaded SLN in-situ gel (T_max_ brain = 0.17 h); The toxicological results indicated the higher safety profile of IN ALM-loaded SLN in situ gel for nasal administration.	[157]
	IN free ALM in-situ gel		6.25 ± 0.03 9.15 ± 0.07	2	1.43 ± 0.02 3.09 ± 0.05	255.1	60.80		
	IV ALM solution		3.32 ± 0.04 12.43 ± 0.09	0.5	1.23 ± 0.02 3.20 ± 0.06	-	-		
Almotriptan malate (ALM)	IN ALM-loaded NLC	Solid lipid: Compritol^®^ ATO 888 Liquid lipid: Labrafil^®^ M 2125 CS Emulsifier(s): Tween^®^ 80 and Lauroglycol	27,291.00 ± 0.02 15,348.60 ± 0.03	0.17	3.44 ± 0.03 1.54 ± 0.02	-	-	Higher C_max_ brain of IN ALM-loaded NLC (7.2-fold vs. IN ALM solution and 6.6-fold vs. oral marketed formulation); Faster onset of IN ALM-loaded NLC (T_max_ brain = 0.17 h); The toxicological results indicated the IN ALM-loaded NLC as safe for nasal administration.	[158]
	IN ALM solution		3387.00 ± 0.05 2541.60 ± 0.05	0.33	0.48 ± 0.04 0.25 ± 0.03	-	-		
	Oral marketed ALM formulation (tablet)		5982.60 ± 0.03 7579.20 ± 0.04	1	0.52 ± 0.05 0.58 ± 0.03	-	-		
Cinnarizine (CIN)	IN CIN-loaded NLC in situ gel	Solid lipid: Cetyl palmitate Liquid lipid: Oleic acid Emulsifier(s): Poloxamer 188 and Soya lecithin	108,000 ± 111.5 41,076 ± 57.46	1	786.65 ± 7.4 345.29 ± 11.2	-	-	Higher C_max_ brain of IN CIN-loaded NLC in situ gel (2.1-fold vs. IN CIN solution).	[159]
	IN CIN solution		48,432 ± 55.81 54,210 ± 81.9	1	380.73 ± 2.41 471.31 ± 7.5	-	-		
Rizatriptan (RZT)	IN RZT-loaded SLN	Solid lipid: Compritol^®^ ATO 888 Emulsifier(s): Tween^®^ 80	1824.82 1894.80	1	583.20 955.18	50.52 *	−97.88 *	Higher C_max_ brain of IN RZT-loaded SLN (1.7-fold vs. IV RZT-loaded SLN and 7.3-fold vs. oral marketed formulation); Faster onset of IN RZT-loaded SLN (T_max_ brain = 1 h); DTE value of IN RZT-loaded SLN not indicated a more effective drug brain targeting after IN administration vs. IV administration.	[160]
	IV RZT-loaded SLN		2375.10 1246.06	2	351.29 175.12	-	-		
	Oral marketed Rizatriptan formulation (tablet)		841.39 −1432.59	4	79.84 103.12	-	-		
Sumatriptan	IN Sumatriptan-loaded NLC	Solid lipid: Stearic acid and Cholesterol Liquid lipid: Triolein Emulsifier(s): Brij^®^ 35 and Brij^®^ 72	0.57 0.19	2	0.18 0.08	258.02	61.23	Higher C_max_ brain of IN Sumatriptan-loaded NLC (9.4-fold vs. IV Sumatriptan-loaded NLC, 5.6-fold vs. IN Sumatriptan solution and 7.4-fold vs. IV Sumatriptan solution).	[161]
	IV Sumatriptan-loaded NLC		0.07 0.06	2	0.02 0.05	-	-		
	IN Sumatriptan solution		0.07 0.10	1	0.03 0.07	-	-		
	IV Sumatriptan solution		0.04 0.36	1	0.02 0.23	-	-		
Zolmitriptan	IN Zolmitriptan-loaded SLN	Solid lipid: Glyceryl monostearate Emulsifier(s): Soya lecithin and Poloxamer 188	0.04 ± 2.45 -	0.5	0.04 ± 1.32 -	-	-	Higher C_max_ brain of IN Zolmitriptan-loaded SLN (2-fold vs. IN Marketed formulation and 2.3-fold vs. IN Zolmitriptan solution); IN Zolmitriptan-loaded SLN showed a higher T_max_ value (0.5 h) due to a slower drug release pattern.	[10]
	IN marketed Zolmitriptan formulation (Zolmist^®^ nasal spray)		0.02 ± 1.65 -	0.17	0.03 ± 2.50 -	-	-		
	IN Zolmitriptan solution		0.02 ± 1.25 -	0.17	0.03 ± 1.56 -	-	-		

AUC_0-t_, area under the curve up to the last quantifiable time-point; C_max_, maximum concentration; DTE, drug targeting efficiency; DTP, drug targeting potential; IN, intranasal; IV, intravenous; NLC, nanostructured lipid carriers; SLN, solid lipid nanoparticles; T_max_, time required to reach the maximum concentration, * Calculated.
